# Reconstructing the Neanderthal brain using computational anatomy

**DOI:** 10.1038/s41598-018-24331-0

**Published:** 2018-04-26

**Authors:** Takanori Kochiyama, Naomichi Ogihara, Hiroki C. Tanabe, Osamu Kondo, Hideki Amano, Kunihiro Hasegawa, Hiromasa Suzuki, Marcia S. Ponce de León, Christoph P. E. Zollikofer, Markus Bastir, Chris Stringer, Norihiro Sadato, Takeru Akazawa

**Affiliations:** 1 Department of Cognitive Neuroscience, Advanced Telecommunications Research Institute International, Kyoto, 619-0288 Japan; 2 Department of Mechanical Engineering, Faculty of Science and Technology, Keio University, Yokohama, 223-8522 Japan; 3 Department of Cognitive and Psychological Sciences, Graduate School of Informatics, Nagoya University, Nagoya, 464-8601 Japan; 4 Department of Biological Sciences, Graduate School of Science, University of Tokyo, Tokyo, 113-0033 Japan; 5 Automotive Human Factors Research Center, National Institute of Advanced Industrial Science and Technology, Tsukuba, 305-8566 Japan; 6 Graduate School of Engineering, University of Tokyo, Tokyo, 113-8656 Japan; 7 Anthropological Institute, University of Zurich, CH-8057, Zürich, Switzerland; 8 Paleoanthropology Group, Department of Paleobiology, Museo Nacional de Ciencias Naturales, 28006 Madrid, Spain; 9 Department of Earth Sciences, Natural History Museum, London, SW7 5BD UK; 10 Department of Cerebral Research, National Institute for Physiological Sciences, Okazaki, 444-8585 Japan; 11 Research Institute, Kochi University of Technology, Kochi, 782-8502 Japan

## Abstract

The present study attempted to reconstruct 3D brain shape of Neanderthals and early *Homo sapiens* based on computational neuroanatomy. We found that early *Homo sapiens* had relatively larger cerebellar hemispheres but a smaller occipital region in the cerebrum than Neanderthals long before the time that Neanderthals disappeared. Further, using behavioural and structural imaging data of living humans, the abilities such as cognitive flexibility, attention, the language processing, episodic and working memory capacity were positively correlated with size-adjusted cerebellar volume. As the cerebellar hemispheres are structured as a large array of uniform neural modules, a larger cerebellum may possess a larger capacity for cognitive information processing. Such a neuroanatomical difference in the cerebellum may have caused important differences in cognitive and social abilities between the two species and might have contributed to the replacement of Neanderthals by early *Homo sapiens*.

## Introduction

The ultimate and proximate causes of the replacement of Neanderthals (NT) by anatomically modern humans remain key questions in paleoanthropology. The disappearance of NT and expansion of *Homo sapiens* have been explained by a number of hypotheses, including differences in ability to adapt to rapidly changing climate and environment^1,2^, differences in technical, economic and social systems^3,4^, differences in subsistence strategies^5,6^, differences in language skill^7^, cannibalism^8^, and assimilation between the two species^9^. Nevertheless, details of the processes leading to replacement are unclear. There is a growing amount of evidence that differences in cognitive or neural function, may help to explain NT replacement by *Homo sapiens*, potentially via behavioural changes resulting from anatomical and functional brain differences^10–16^. However, although shape differences in braincases (endocasts) were reported^11–14^, there are no studies of antemortem brain reconstruction from the fossil crania to infer possible functional differences between the two species. Here, we present a detailed virtual reconstruction of the brains of NT and early *Homo sapiens* (EH) using computational anatomy, in order to infer possible morphological differences in the brain between the two species. The brain structure of chimpanzees (*Pan troglodytes*) and bonobos (*Pan paniscus*) has comparable anatomies^17^ and morphing of chimpanzee brains can sufficiently reconstruct bonobo brains (and vice versa) (See Methods), despite the fact that divergence between the two species is considered to have occurred approximately 1.5–2.1 million years ago^18^. Since the divergence between the NT and anatomically modern humans took place much more recently (approximately 0.6–0.8 million years ago^19^), we can reasonably predict the fossil brains by deforming the modern *Homo sapiens* (MH) brains.

Computed tomography (CT) scan data of four adult NT [Amud 1 (~50,000–70,000 years old^20,21^), La Chapelle-aux-Saints 1 (~47,000–56,000 years old^22^), La Ferrassie 1 (~43,000–45,000 years old^23^) and Forbes’ Quarry 1 (no dating information)] and four EH [Qafzeh 9 (~90,000–120,000 years old^22,24,25^), Skhul 5 (~100,000–135,000 years old^26,27^), Mladeč 1 (~35,000 years old^28^) and Cro-Magnon 1 (about 32,000 years old^29^)] were obtained. Three-dimensional (3D) endocranial surface models were generated using conventional virtual anthropology techniques (see Methods). Next, we reconstructed the brain morphology of each fossil cranium based on computational anatomy image processing techniques. In brief, the 3D structure of the brain (grey and white matter regions) and endocast (brain and cerebrospinal fluid regions) were obtained by segmenting 1,185 cranial MRI scans from living humans based on a probabilistic framework using Statistical Parametric Mapping (SPM) software (http://www.fil.ion.ucl.ac.uk/spm/). The shapes of the population average endocast and the brain were then calculated. The spatial deformation function from each of the endocasts to the average endocast was defined using a diffeomorphic spatial deformation (DARTEL) algorithm^30^. The 3D structure of the brain enclosed in the fossil cranium was computationally reconstructed by deforming the average human brain using the deformation function from the average endocast to each fossil endocast (see Methods). The volume of each brain region was also estimated using the neuroanatomical labels for the brain locations, which is quite impossible just by analysing and quantifying the fossil endocranial surfaces (see Methods).

In the present study, we did not transform each individual brain but the averaged brain was transformed to reconstruct the fossil brains. If we used the brain model of one subject to estimate the brain model of another subject based on endocranium, the estimation accuracy of each brain region was found to be relatively low since inter-individual variability of the sulcal and gyral morphology in the human brain is large. However, if we averaged human brain images using the DARTEL algorithm for estimation of the brain morphology, the estimation accuracy was much improved because the estimated brain was less affected by subject-specific sulcal and gyral patterns and better represented overall general structure of the brain (See Methods).

## Results and Discussion

**Figure 1:**
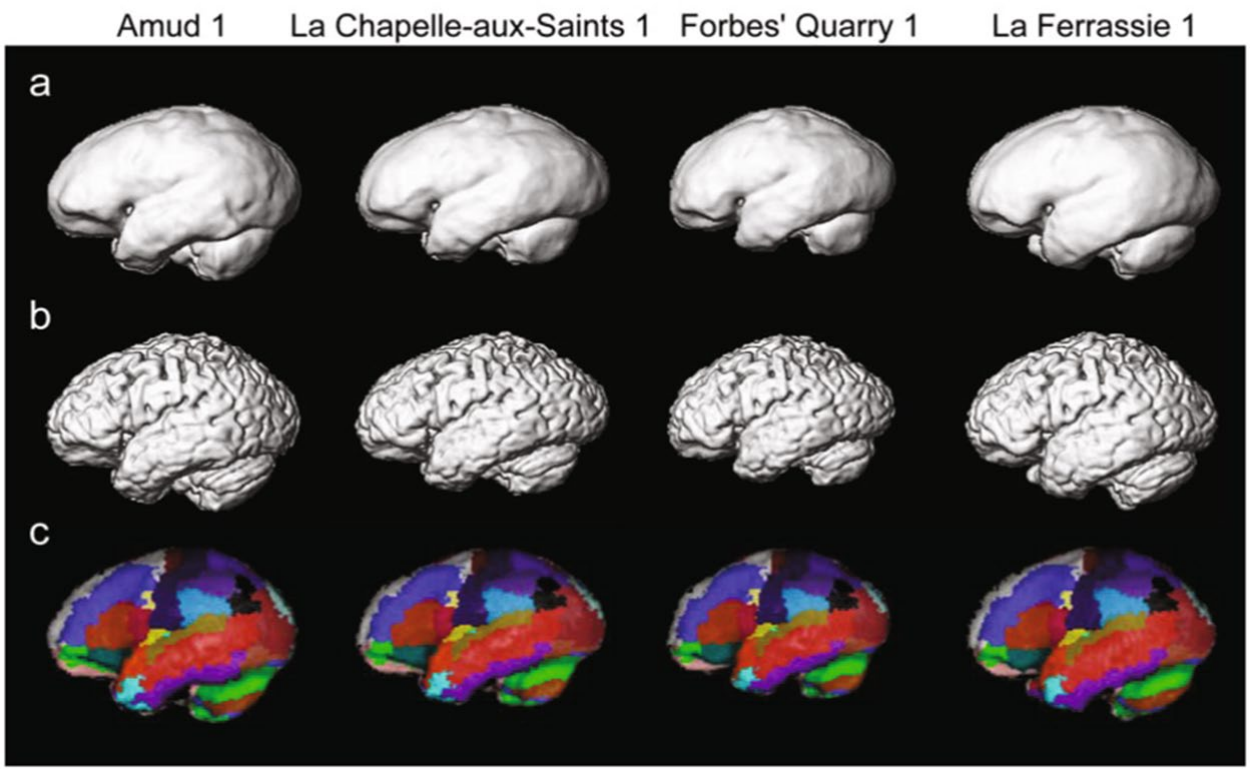
Reconstructed Neanderthal brains. (**a**) Population-average. (**b**) Representative modern human subject. (**c**) The reconstructed brains with the neuroanatomical labels.

The reconstructed brains from four NT crania are shown in Fig. 1. The brains were reconstructed using the population-averaged modern human brain (upper panel) or from one representative modern human brain (middle panel). The reconstructed brains with the neuroanatomical labels were also presented (lower panel). Identifying cortical features such as imprints of sulci and gyri on the endocranial surface (and placing landmarks) is actually very difficult since such imprints are very subtle on human and Neanderthal fossil crania. However, here we assumed that modern human brain maps are the best available and most parsimonious proxy of the brain morphology of the last common ancestor of humans and Neanderthals, and the location of brain regions was predicted from the NT and EH endocasts. Therefore, using the present reconstruction method, the position and shape of the sulci and gyri can be well estimated, allowing more detailed, unbiased investigations of the brain morphology.

The cerebral and cerebellar volumes of the reconstructed NT and EH brains were 1304 and 182 cc for Amud 1; 1159 and 140 cc for La Chapelle-aux-Saints 1; 1268 and 166 cc for La Ferrassie 1; 912 and 106 cc for Forbes’ Quarry 1; 1075 and 147 cc for Qafzeh 9; 1053 and 146 cc for Skhul 5; 1205 and 165 cc for Mladeč 1; and 1208 and 156 for Cro-Magnon 1, respectively (See Method). The mean (±standard deviation) cerebral and cerebellar volumes of NT, EH and MH were 1161 ± 177 cc and 149 ± 33 cc, 1135 ± 83 cc and 153 ± 9 cc, and 1097 ± 115 cc and 149 ± 15 cc, respectively. No statistically significant between-group difference was detected in the total brain volume. However, the mean ratios of cerebellum to cerebrum in NT, EH and MH were 0.127 ± 0.010, 0.135 ± 0.004 and 0.136 ± 0.005, respectively. One-way ANOVA with multiple comparisons (Ryan’s method) indicated that NT had significantly smaller relative cerebellar volume than EH and MH (F_*2*,1190_ = 8.53, *p* < 0.001, NT vs. EH *t*_1190_ = 2.47, *p* < 0.05, NT vs. MH *t*_1190_ = 4.09, *p* < 0.05, respectively).

**Figure 2:**
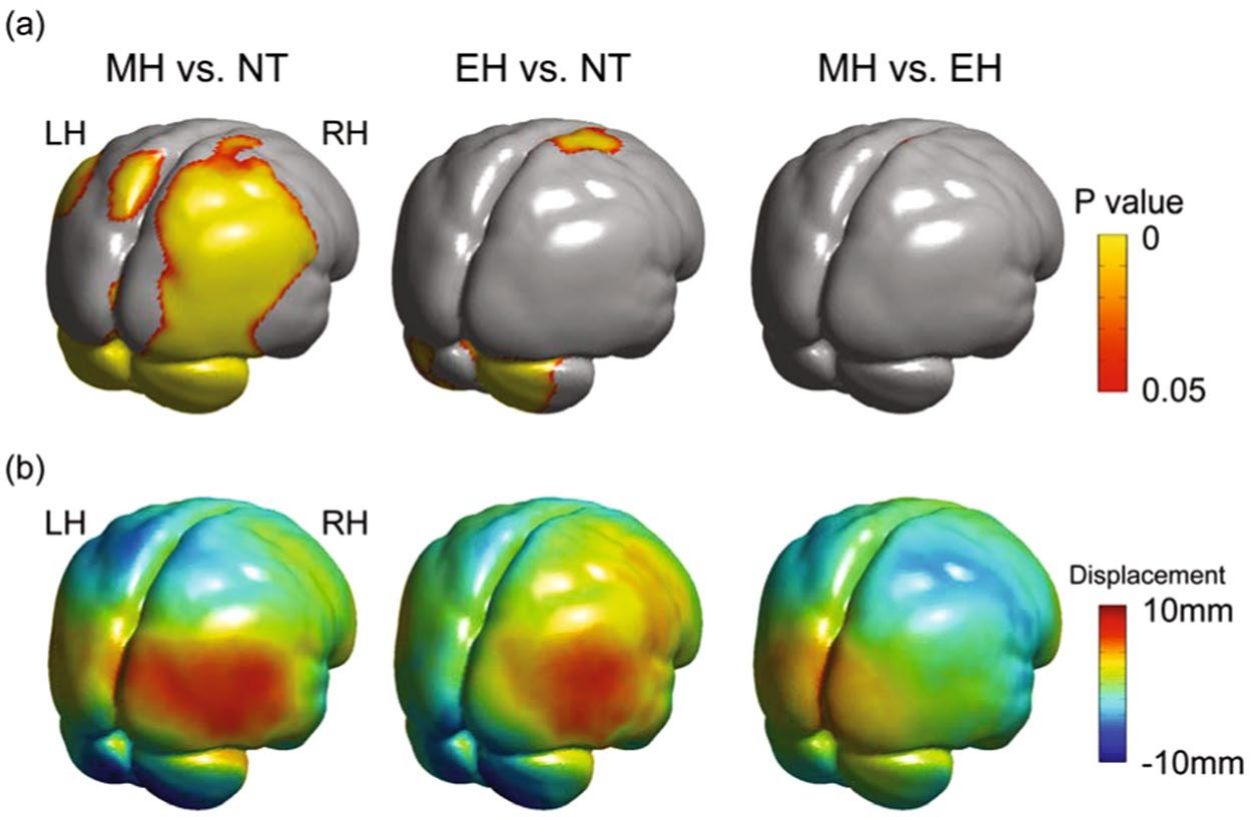
Comparisons of the brain surface morphology among Neanderthal, early *Homo sapiens* and modern *Homo sapiens*. (**a**) Surface statistical map shows the surface area where the differences are statistically significant (*p* < 0.05 with family-wise error (FWE) correction). See Extended Data Fig. 1 for more details. (**b**) Surface displacement maps show the morphological difference in the direction perpendicular to the tangential surface. The displacement maps were calculated by subtracting modern *Homo sapiens* (MH) from Neanderthal (NT), early *Homo sapiens* (EH) from NT, and MH from EH, respectively. See Extended Data Fig. 2 for more details.

The surface morphology between NT, EH and MH was compared using a surface displacement-based morphometry (Fig. 2) (See Method). There were significant morphological differences in the cerebellar, parietal, occipital and medial temporal regions, but no differences in the frontal regions between NT and MH (Fig. 2(a) NT vs MH). Between NT and EH, there were significant differences in the cerebellar and part of the right medial temporal and right somato-motor regions (Fig. 2(a) NT vs EH). Virtually no difference was observed between MH and EH, except for small part of the right somato-motor region (Fig. 2(a) MH vs EH). Significant difference was also noted in the basilar region, but this occurred possibly due to complex morphology of the sphenoid bone. Therefore, the largest morphological difference between NT and the EH-MH lineage was observed in the cerebellar hemisphere, which was significantly more inferiorly projected in EH and MH than in NT (Fig. 2).

**Figure 3:**
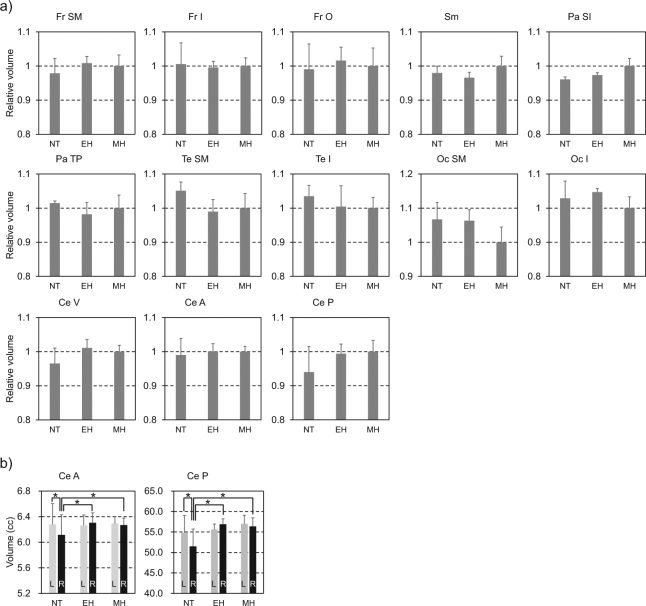
Comparisons of the relative volumes of the parcellated brain regions among NT, EH and MH. (**a**) Each parcellated volume was normalized to the mean MH volume to calculate a ratio (i.e. relative volume unit). The regionally specific volume differences were evaluated after removing the effects of ICV by analysis of variance (ANOVA) across 13 parcellated regions. We employed Bonferroni correction for multiple comparisons, so that the threshold of *p* < 0.003 (=0.05/13) is set to statistically significant. The relative volume differences were found in Pa SI (F_*2,1190*_ = 9.31, *p* = 0.0001), Oc SM (F_*2,1190*_ = 8.15, *p* = 0.0003), Ce V (F_*2,1190*_ = 7.34, *p* = 0.0007), and Ce P (F_*2,1190*_ = 6.70, *p* = 0.0013) (Extended Data Table 3). The mean (±standard deviation) MH volumes of the parcellated brain regions are 161.61 ± 5.22 cc for Fr SM, 41.57 ± 1.00 cc for Fr I, 65.46 ± 3.45 cc for Fr O, 96.93 ± 2.82 cc for Sm, 88.30 ± 1.98 cc for Pa SI, 37.38 ± 1.41 cc for Pa TP, 91.27 ± 3.92 cc for Te SM, 82.50 ± 2.60 cc for Te I, 93.70 ± 4.17 cc for Oc SM, 39.99 ± 1.32 cc for Oc I, 12.38 ± 0.23 cc for Ce V, 13.86 ± 0.21 cc for Ce A, and 114.41 ± 3.76 cc for Ce P. (**b**) As the ANOVA results indicated a significant group-by-laterality difference in the size-adjusted volume of the cerebellar hemisphere (F_*2,1190*_ = 14.28, *p* < 0.001 for Ce A, F_*2,1190*_ = 12.73, *p* < 0.001 for Ce P), we tested if there is a significant difference between the size-adjusted volume of the left and right cerebellar regions within each group and between groups based on the symmetrized volume analysis. Fr, frontal lobe; Pa, parietal lobe; Te, temporal lobe; Oc, occipital lobe; Ce, cerebellum; Sm, sensorimotor cortex; SM, superior and middle region; I, inferior region; O, orbitofrontal region; SI, superior and inferior region; TP, temporo-parietal junction; A, anterior region; P, posterior region; V, vermis. *p < 0.05 corrected for multiple comparisons. Data are means ± s.d. See Extended Data Table 1 for correspondence between the automated anatomical labelling (AAL) atlas and the parcellated brain regions.

The volume of each parcellated region between the groups was also compared (Fig. 3) after adjustment for total intracranial volume (ICV) was performed by including ICV as a covariate in a linear model (ANCOVA) and regressing it out^31^. This size adjustment is necessary to correct for large interindividual variability in the geometrical size of the specimens used in the present study. The results of analysis of variance showed that the size-adjusted volume differences among three groups were found in the superior and inferior region of parietal lobe, occipital regions, and cerebellum (Fig. 3). As we conducted post hoc test between 3 groups, only the cerebellum has a significant difference both between NT and EH (*t*_1190_ = 3.41, *p* < 0.05 corrected for multiple comparisons with Ryan’s method, for cerebellar vermis, and *t*_1190_ = 2.33, *p* < 0.05 for posterior cerebellar hemispheres) and NT and MH (*t*_1190_ = 3.64, *p* < 0.05, and *t*_*1190*_ = 3.64, *p* < 0.05, respectively). Namely, the size-adjusted volume of the cerebellum (vermis and posterior hemispheres) was significantly larger in the EH–MH lineage.

Our results of the surface displacement-based morphometry and the comparison of the size-adjusted volume of each parcellated region indicated that the cerebellum has the most prominent morphological and volumetric difference between NT and the EH-MH lineage. Previously, Weaver suggested that the cerebellum was relatively larger in MH than in terminal Pleistocene humans, and the enlargement of the cerebellum in the MH lineage started to occur sometime after 28,000 years ago^14^. However, this study clearly indicated that the cerebellum started to enlarge in the EH-MH lineage far before the time when NT disappeared because the relative cerebellar volume was much larger than NT not only in Mladeč 1 and Cro-Magnon 1, but also in Qafzeh 9 and Skhul 5.

There is now strong evidence that the cerebellar hemispheres are important for both motor-related function and higher cognition including language, working memory, social abilities and even thought^32–34^. Further, whole cerebellar size is correlated with cognitive abilities, especially in the verbal and working memory domain^35^. Thus, we examined the relationship between cerebellar volumes and various cognitive task performances using a large data set from the human connectome project (see Methods). Multiple regression analyses revealed that attention and inhibition task score was most strongly correlated with size-adjusted whole cerebellar volumes (*t*_1090_ = 4.27, *p* < 0.001), followed by cognitive flexibility task score (*t*_1090_ = 3.24, *p = *0.001). There was also a significant correlation of size-adjusted cerebellar volumes with speech comprehension (*t*_1090_ = 3.33, *p* = 0.001), speech production (*t*_1090_ = 2.86, *p* = 0.004), working memory (*t*_1090_ = 2.92, *p* = 0.004), episodic memory (*t*_1090_ = 2.84, *p* = 0.005) task scores, but not with processing speed task score (*t*_1090_ = 1.29, *p* = 0.199). Note that the functions such as attention, inhibition, cognitive flexibility, working memory, are thought to be main components of executive functions^36^. These results indicate that the cerebellar hemispheres are involved in the abilities of executive functions, language processing, and episodic memory function.

Unlike complex neuronal networks in the cerebrum, the cerebellar neural circuit (module or microcomplex) is anatomically simple and uniform^32^. As the cerebellar hemisphere contains many of these modules^37^, a larger cerebellar volume is directly correlated with larger number of the modules, and therefore with higher language processing and larger working memory capacity. Language processing refers to the ability to produce and comprehend sounds and signs, which enables shared communication between individuals^38^. Working memory is a temporary memory storage and executive information processing system used for cognitive abilities such as learning and reasoning^39^. In addition, these functional modules can encode essential properties of mental representation in the cerebrum for various cognitive activities^40^, possibly leading to the correlation between the size-adjusted cerebellar volume and the ability of executive functions. Thus, *Homo sapiens* with relatively larger cerebellar hemispheres may possess higher cognitive and social functions.

Furthermore, we noticed that there seems to exist a possible evidence for bilateral volumetric asymmetry in the NT cerebellum but not in the cerebellum of the EH-MH lineage. To examine the volumetric laterality of the cerebellum within a group, we recalculated the volume of each cerebellar region using a symmetrized automated anatomical labelling (AAL) atlas as well as a mirror of this symmetrized atlas (See Methods). We found that the right side of anterior and posterior cerebellum was significantly smaller than that of the left in NT (simple main effect F_*1,119*0_ = 34.85, *p* < 0.001, and F_*1,1190*_ = 26.44, *p* < 0.001, respectively) but no statistical differences were detected in EH (F_*1,1190*_ = 2.24, *p* = 0.13, and F_*1,1190*_ = 3.84, *p* = 0.05, respectively) and MH (F_*1,1190*_ = 0.77, *p* = 0.38 and F_*1,1190*_ = 0.99, *p* = 0.32, respectively). We also found that the relative volume of the right cerebellar hemisphere was significantly smaller in NT compared with that in EH (*t*_2380_ = 2.41, *p* < 0.05 corrected for multiple comparisons with Ryan’s method for anterior cerebellar hemisphere, and *t*_2380_ = 3.70, *p* < 0.05 for posterior cerebellar hemisphere) and MH (*t*_2380_ = 2.77, *p* < 0.05 and *t*_2380_ = 4.74, *p* < 0.05, respectively), with no differences between EH and MH and no differences in the left hemisphere among the three groups.

The functions of the cerebellar hemispheres differ according to location, as different parts of the cerebellum are anatomically and functionally connected to different regions of the cerebrum^41^. In particular, the lateral parts of the cerebellar hemisphere are anatomically connected to the opposite side of the association cortices in the cerebrum^42^. Our finding of laterality in terms of the relatively small right cerebellar hemisphere of NT indicates minimal connection to the left prefrontal regions, which has one of the major role in language processing^38^, potentially causing disparity of language ability between NT and *Homo sapiens*. However, the preservation in the cerebellar region of the fossils is certainly not perfect and there might be asymmetry related to taphonomy in addition to the innate morphological asymmetry in the region. Therefore, morphological laterality of the Neanderthal cerebellum needs to be confirmed in future studies with a large number of cases.

In the present study, the MH had relatively larger parietal regions than the NT with significant difference, particularly in the superior medial and lateral areas (Figs 2 and 3), as suggested by Bruner *et al*.^11^. However, there were no differences in the relative size of the parietal region between NT and EH. The superior medial part of the parietal lobule (the precuneus) plays important roles in highly integrated tasks, including visuo-spatial imagery, episodic memory and self-related mental representations^43^, whereas the superior lateral region is involved in integration and coordination between the self and the external space, generation of body image and sense of agency^44^. In addition, the parietal regions have strong connections to the cerebellar hemispheres and the frontal cortex^41^. These findings indicate that enlargement of this region in MH may have improved cognitive function in harmony with the cerebellar hemispheres and the frontal region.

Previous studies have reported significant differences in the occipital and medial temporal regions between NT and *Homo sapiens*. Pearce *et al*. estimated that NT had larger visual cortices than EH based on the orbit size of fossil crania^45^. In support, the occipital region was significantly larger in NT than in EH in the present study (Figs 2 and 3). There are also reported differences in the basicranial morphology between NT and EH, with NT having a relatively narrower orbitofrontal cortex, smaller olfactory bulbs and less increased and forward-projecting temporal lobe poles^12^. We also observed clear differences in basicranial morphology between the two species. The anterior part of the medial temporal region was more inferiorly projected in NT than in EH and MH (Fig. 2), which is consistent with data from Bastir *et al*.^12^ showing a relatively low temporal pole position in NT.

In the present study, we used the average human brain to reconstruct NT and EH brains. Therefore, the variation within NT or EH was basically estimated based on four fossil brains. However, to account for possible larger variation within NT or EH, we also reconstructed 4 × 1185 NT and 4 × 1185 EH brains, assuming that the variation within NT and EH was equivalent to that of MH. The intra- and inter-specific variation in each parcellated brain region was analysed and evaluated based on the Cohen’s d effect size and statistical test. We confirmed that our results are not affected by the use of the averaged brain for our reconstructions (See Extended Data Fig. 4).

In conclusion, we found that NT had significantly relatively smaller cerebellar hemispheres than *Homo sapiens*, particularly on the right side. Larger cerebellar hemispheres were related to higher cognitive and social functions including executive functions, language processing and episodic and working memory capacity. Based on archaeological records, Wynn and Coolidge suggested that NT had a smaller capacity of working memory^46^, which is also related to the capacity for cognitive fluidity proposed by Mithen^47^. Moreover, such differences in the capacity for cognitive fluidity were hypothesized to mainly originate from language processing ability^48^. Thus, the differences in neuroanatomical organization of the cerebellum may have resulted in a critical difference in cognitive and social ability between the two species. Consequently, ability to adapt to changing environment by creating innovation may have been limited in NT and this difference possibly affected their chance of survival and drove the replacement process.

## Materials and Methods

### Fossil specimens and 3D reconstruction

Three-dimensional (3D) endocranial surface models were generated using conventional virtual anthropology techniques^49–52^. CT scan data of four adult NT (Amud 1, La Chapelle-aux-Saints 1, La Ferrassie 1 and Forbes’ Quarry 1) and four anatomically modern humans (Qafzeh 9, Skhul 5, Mladeč 1 and Cro-Magnon 1) were obtained and 3D endocranial surface models were generated as triangular mesh models based on the marching cube method using Analyze 9.0 (Biomedical Imaging Resource, Mayo Clinic, Rochester, MN, USA). For the Amud 1 cranium, the fragments comprising the fossil cranium were separated by virtually removing the adhesive and plaster, and these fragments were mathematically reassembled based on the smoothness of the joints^53,54^. To restore missing portions of a NT cranium, other NT crania were warped onto the target cranium using an iterative thin-plate spline deformation. Specifically, we defined 185 conventional anatomical and sliding semi-landmarks on the endocranial surface, and using common existing landmarks, the warping (thin-plate spline) function from one cranium to the other was calculated. The cranium of Forbes’ Quarry 1 was warped onto that of La Chapelle-aux-Saints 1 to reconstruct the damaged basicranial regions of La Chapelle-aux-Saints 1, and this reconstructed cranium was then warped onto the Amud 1. The cranium of La Chapelle-aux-Saints 1 was warped onto both Forbes’ Quarry and La Ferrassie 1 crania for respective reconstructions. Finally, any remaining small holes on the endocranial surfaces were restored either by warping a human cranium or by a gap-filling algorithm. As the crania of the anatomically MH were comparatively better preserved, we interpolated missing regions by only warping a human cranium or by a gap-filling algorithm. See refs^54,55^ for further information on the reconstruction procedure of fossil endocasts.

### Reconstruction of fossil brains

To reconstruct the brain morphology of fossil hominins based on the reconstructed endocasts, we obtained high-resolution T1-weighted MR data of a total of 1,185 living humans from the IXI Dataset (http://brain-development.org/ixi-dataset/) (87 women and 98 men, age range: 20–40 years), the Human Connectome Project (http://www.humanconnectome.org/) (291 women and 197 men, age range: 22–35 years) and National Institute for Physiological Science Japan (256 women and 256 men, healthy Japanese volunteers, age range: 18–46 years; the protocol was approved by the ethical committee of the National Institute for Physiological Sciences, Okazaki, Japan, and the ethical committee of Faculty of Science and Technology, Keio University, Yokohama, Japan, all methods were performed in accordance with relevant guidelines and regulations, and all participants provided their written informed consent). We combined the above three datasets to account for large intraspecific variability in the sulcal and gyral patterns in the modern human brain. Using the SPM software package, grey matter (GM), white matter (WM) and cerebrospinal fluid (CSF) regions were segmented using the unified segmentation-normalization procedure^56^, and the 3D structure of the human brain (GM + WM) and the corresponding endocast (GM + WM + CSF) were obtained.

The 3D structure of the fossil brain was computationally reconstructed by deforming the human brains. Specifically, the shape of the population average endocast was calculated based on 1,185 human and eight fossil endocasts. The spatial deformation function from each of the endocasts to the average endocast was defined using a DARTEL algorithm, such as to minimize the mean squared difference between the images and the linear elastic energy of the deformation field (the DARTEL algorithm is a diffeomorphic mapping, hence the transformation can be inverted). This deformation function was then used to transform each brain enclosed in the endocast to calculate the population-average brain. To reconstruct the fossil brain of the NT and EH, the average human brain was finally transformed back to the fossil endocasts using the inverse of the above-defined deformation function.

### Calculation of the cerebral and cerebellar volumes

To quantify the volumes of the cerebrum and cerebellum of the reconstructed fossil brain, the representative modern human brain^57^ was parcellated into 40 anatomical regions using FreeSurfer software^58^. The resulting parcellated brain was then inversely transformed to the fossil endocasts as described previously and the volumes were measured. The cerebral and cerebellar volumes were defined as the sum of GM and WM volume of the cerebrum and cerebellum, respectively. For size adjustment, total intracranial volume (ICV) was calculated as the sum of the GM, WM and CSF volumes.

### Evaluation of the reconstructed brain surface

To evaluate the accuracy of the present brain reconstruction method, we reconstructed MH brains based solely on endocast information. The average human brain was transformed based on the deformation function from the average endocast to each individual human endocast, and then compared with the corresponding true brains. Spatial distribution of Euclidean deviation from the true brain surface to the estimated surfaces was quantified (Extended Data Fig. 3). A comparatively larger deviation was observed in the superior parietal lobule, although the mean absolute deviation was relatively small (1.81 ± 0.58 mm).

### Evaluation of the parcellated brain volumes

We also evaluated the accuracy of the estimated volume of each of the parcellated brain region using the automated anatomical labelling (AAL) technique^57^. We parcellated each individual brain into 25 structural regions (12 per hemisphere and cerebellar vermis) based on the AAL atlas (Extended Data Table 1), and then compared each region of the true and estimated brains by counting the number of true positive (TP), true negative (TN), false positive (FP) and false negative (FN) voxels. The accuracy of the volume estimation of each parcellated region was calculated as the ratio between the number of correctly identified voxels (TP + FN) and the total number of voxels (TP + FN + TN + FP) in the corresponding brain region. The mean accuracy of each region was larger than 88%, except for the sensorimotor region (Extended Data Table 2), indicating that the present brain reconstruction method had sufficient accuracy for estimating the volume of each parcellated brain region. On the other hand, if we used the brain model of one subject to estimate the brain model of other subjects, the estimation accuracy of each brain region was much reduced [in the range of 65 (sensory-motor regions) to 90% (cerebellar regions), based on all possible combinations of 30 MRI data (30 × 29 = 870 combinations; 10 random sampling data from each dataset)].

### Surface displacement-based morphometry

To evaluate differences in surface morphology of the brains, the surface mesh model of the average brain (with approximately 50,000 vertices) was transformed back to the individual brains using the inverse deformation functions. After Procrustes superimposition and size normalization, the morphological differences between each individual and the average brain surfaces were quantified in the form of a 3D displacement field, and the differences in the displacement fields were statistically tested among NT, EH and MH groups by multivariate analysis of variance. The surface statistical map was generated after computing Hotelling’s T-square statistics on a vertex-by-vertex basis. This is possible because the average brain surface mesh model was transformed back to calculate all the brain surfaces; hence the mesh models are all homologous. The surface statistical map was thresholded at *p* < 0.05 family-wise error (FWE) corrected for multiple comparisons by means of random field theory^59^ using the Surfstat toolbox (http://www.math.mcgill.ca/keith/surfstat/).

### Correlation between cognitive abilities and cerebellar volume

We analysed data from the open dataset of the WU-Minn Human Connectome Project^60^. Specifically, we used defaced T1-weighted whole-brain structural images and performance across various cognitive domains measured with the psychological test battery in the NIH toolbox reported in the open HCP dataset. For the present analysis, we used data from 1095 participants (500 men and 595 women, 22–36 years of age). The cerebellar grey matter volume was obtained by summing the volume of 26 cerebellar regions distinguished by the AAL atlas. The psychological test battery evaluates the ability of language comprehension and production processing, working memory, cognitive flexibility, attention control, processing speed and episodic memory^61,62^. Finally, we performed multiple regression analyses using each task score as an independent variable and total ICV, age, sex as confounding variables. Regression coefficient for each task score was evaluated by t-statistics with 1090 degrees of freedom. We employed Bonferroni correction for multiple comparisons over 7 regression models, so that the threshold of *p* < 0.007 (=0.05/7) is set to statistically significant.

### Evaluation of the volume laterality in the cerebellum

To evaluate the laterality of the cerebellar volume, we recalculated the volume of each cerebellar region using a symmetrized AAL atlas. Specifically, both original and midsagitally-flipped individual brain images were spatially transformed using a DARTEL algorithm to calculate the symmetrized population-averaged brain shape. The estimated deformation function was used to calculate the symmetrized atlas. However, perfect symmetrisation of the atlas was impossible. Therefore, we used both the symmetrized and the mirror of the symmetrized ALL atlases for parcellation of the cerebellar regions and both data were combined to test if there is a significant difference between the size-adjusted volume of the left and right cerebellum.

### Evaluation of the reconstruction method using chimpanzee and bonobo brains

We tried to reconstruct bonobo brains from chimpanzee brains, and vice versa, based on the open source MRI data of 8 chimpanzees and 3 bonobos (obtained from the National Chimpanzee Brain Resource (NS092988); http://www.chimpanzeebrain.org/) using essentially the same, but not identical technique. The only difference is that FAST (FMRIB’s Automated Segmentation Tool) algorithm^63^ in FSL software (https://fsl.fmrib.ox.ac.uk/fsl/fslwiki/FAST) was used for segmentation of the brains, instead of the unified segmentation-normalization procedure^56^ in SPM software. This is because SPM requires tissue probability maps (TPMs) as the priors, there are no publically available TPMs for chimpanzee and bonobo brains, and we could not successfully generate TPMs by ourselves because of insufficient number of the images. For evaluation, partial distribution of Euclidean deviation from the bonobo brain surface to the estimated surface using the chimpanzee and from the chimpanzee brain surface to the estimated surface using the bonobo was quantified (Extended Data Fig. 5b). The mean (±standard deviation) absolute deviation over the entire brain surface was confirmed to be relatively small (2.1 ± 0.5 mm), indicating the reconstruction of a bonobo brain from a chimpanzee based on the endocast morphology works quite well, and the same method can possibly be applied for the reconstruction of Neanderthal and early *Homo sapiens* brains.

### Data availability

Regarding the 1,185 MR data of living humans, the data from IXI Dataset (http://brain-development.org/ixi-dataset/) and the Human Connectome Project (http://www.humanconnectome.org/) are available from the websites. The data from National Institute for Physiological Sciences are available from the corresponding authors on reasonable request. The data for the correlation analysis are available from the open HCP dataset (http://www.humanconnectome.org/).

## Electronic supplementary material

Supplementary Information
