# Supplementary material for: Reconstructing the Neanderthal brain using computational anatomy
Source: Sci Rep. 2018 Apr 26;8:6296. doi: 10.1038/s41598-018-24331-0 (PMC5919901; doi:10.1038/s41598-018-24331-0)
Supplement: Supplementary file 1 — Supplementary Information [file 41598_2018_24331_MOESM1_ESM.pdf]

Reconstructing the Neanderthal brain using computational anatomy

Takanori Kochiyama, Naomichi Ogihara, Hiroki C. Tanabe, Osamu Kondo, Hideki Amano, Kunihiro Hasegawa, Hiromasa Suzuki, Marcia S. Ponce de León, Christoph P. E. Zollikofer, Markus Bastir, Chris Stringer, Norihiro Sadato & Takeru Akazawa

Supplementary Information

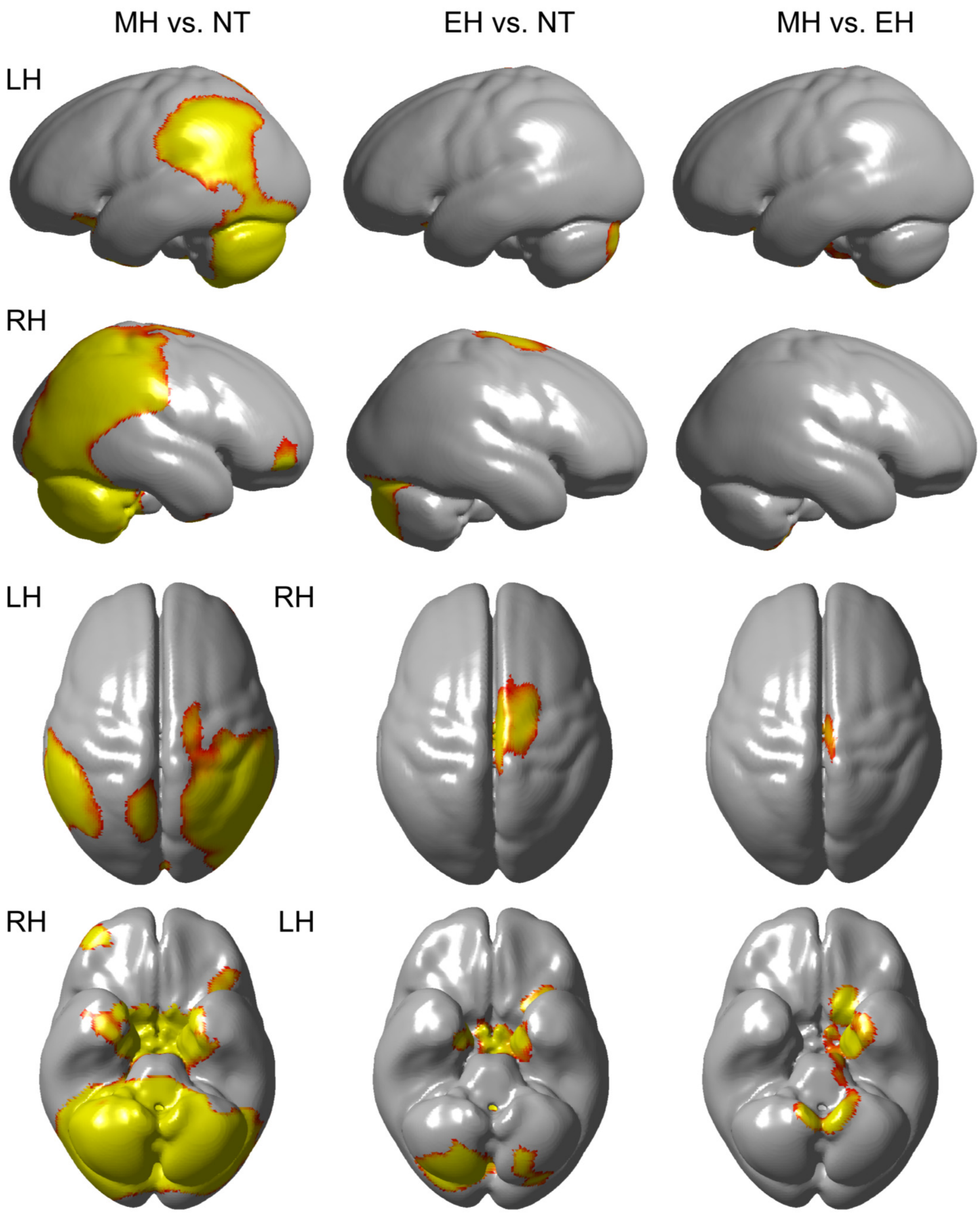

Extended Data Figure 1 | Comparisons of brain surface morphology among NT, EH and MH. The surface statistical map shows the surface area with statistically significant differences ($p < 0.05$, FWE-corrected).

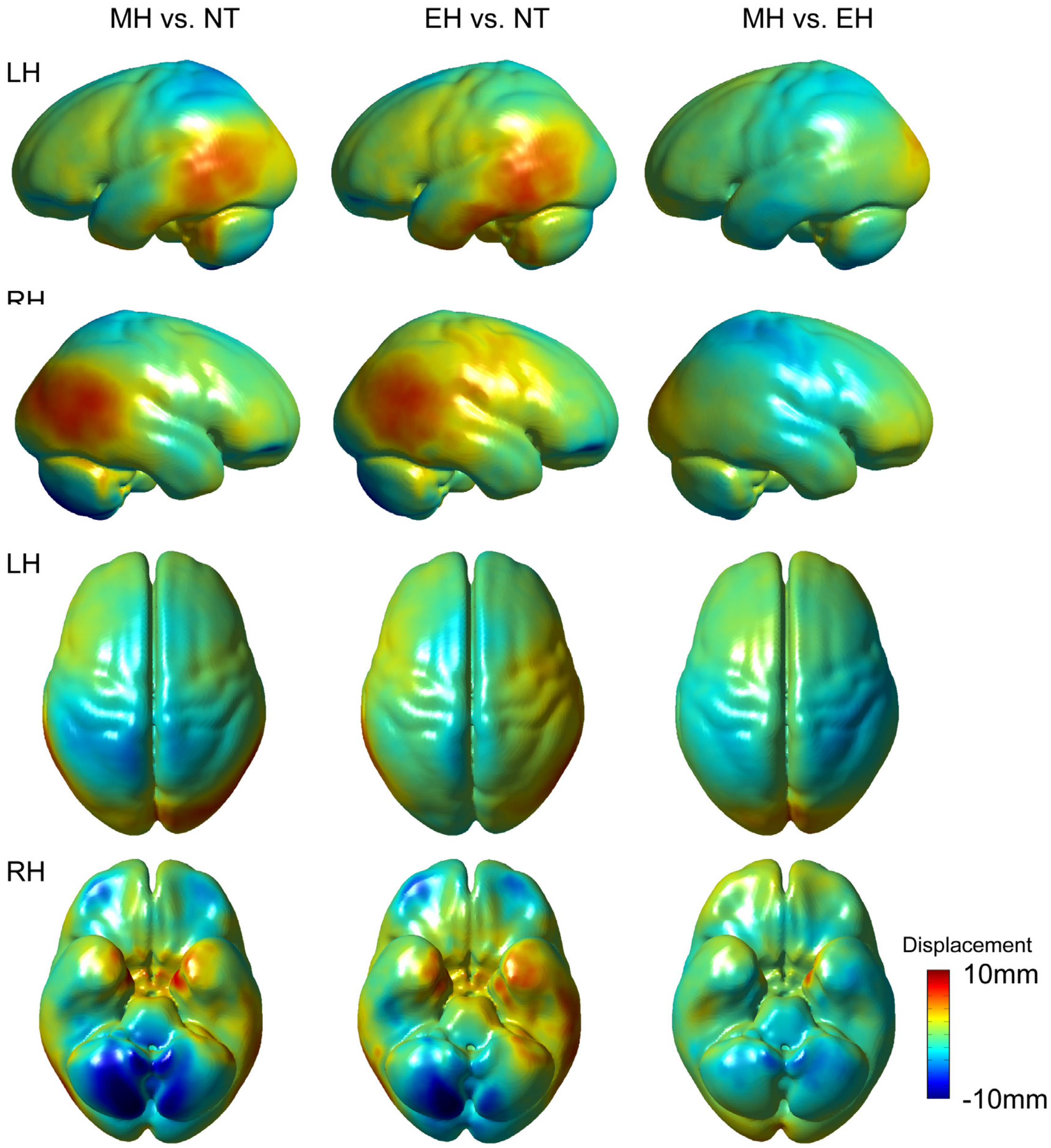

Extended Data Figure 2 | Comparisons of brain surface morphology among NT, EH and MH. The surface displacement maps show the morphological difference in the direction perpendicular to the tangential surface. The displacement maps were calculated by subtracting MH from NT, EH from NT, and MH from EH, respectively.

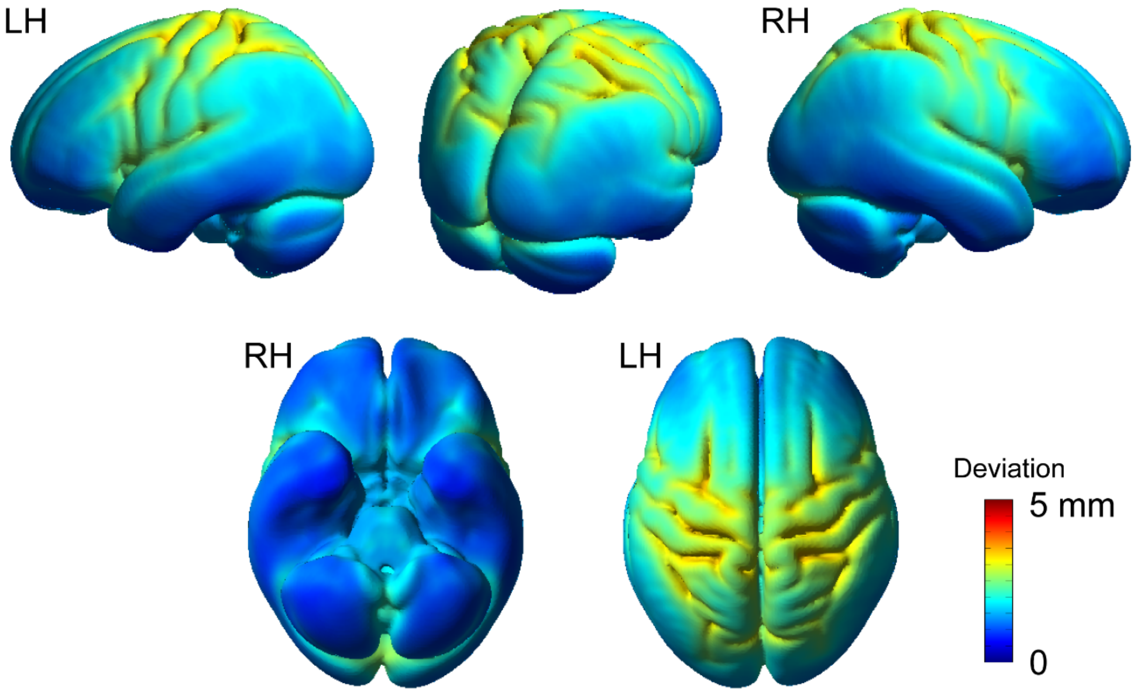

Extended Data Figure 3 | Evaluation of brain reconstruction accuracy. The colour map indicates the degree of accuracy.

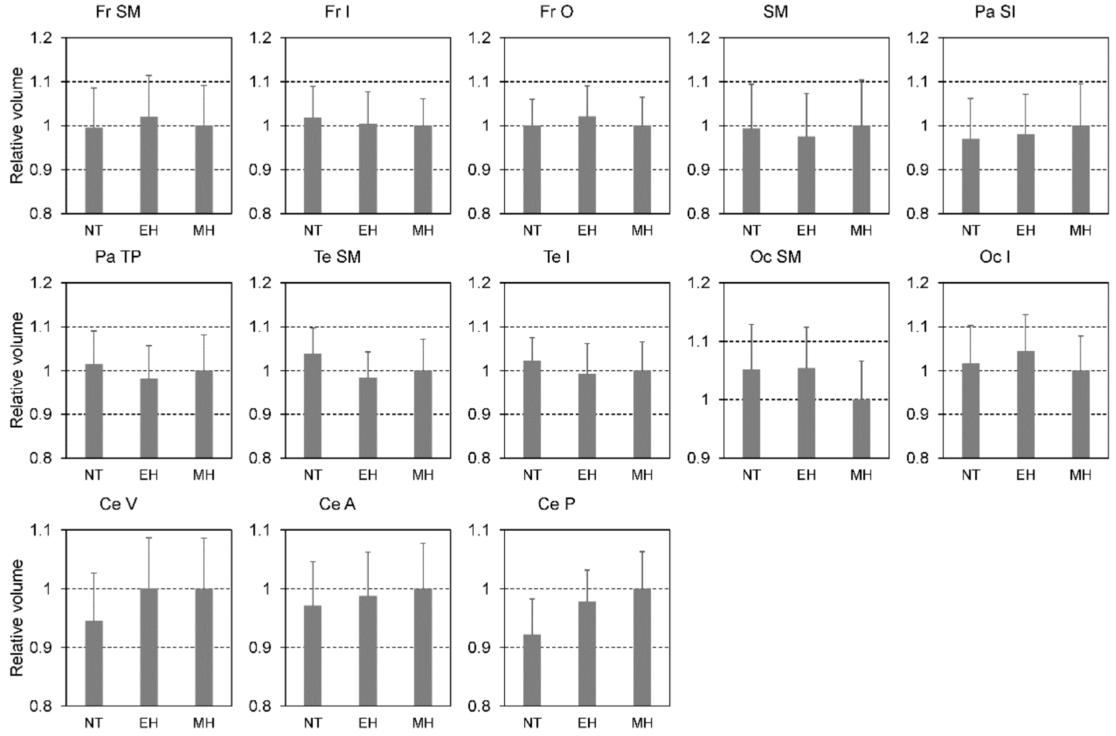

Extended Data Figure 4 | Comparisons of the relative volumes of the parcellated brain regions among NT, EH and MH, assuming that the variation within NT and EH was equivalent to that of MH. To account for possible large variation in the volume of the parcellated brain regions within NT or EH, each NT and EH brain was reconstructed from 1185 individual MH brains instead of the average human brain. Therefore, 4 × 1185 NT, 4 × 1185 EH and 1185 MH brains were used to estimate the regionally specific volume for the 13 parcellated brain regions. Each parcellated volume was normalized to the mean MH volume to calculate a ratio (i.e. relative volume unit). The general pattern of the inter-specific variation shown here is essentially similar to that shown in Figure 3 based on the averaged-brain based reconstruction. However, the intra-specific variation represented by the standard deviation here is much larger than that in Figure 3 because the variation within NT and EH was emulated by that of MH in this individual brain-based reconstruction. The intra- and inter-specific variation in each parcellated brain region was evaluated based on the Cohen's d effect size and statistical test under the assumption that the variation within NT and EH was equivalent to that of MH. The posterior cerebellar volumes were found to be significantly smaller in NT than in EH and MH as in Figure 3, with large effect size (Cohen's d = 0.90 and $p < 0.001$ for NT vs. EH and 1.23 and $p < 0.001$ for NT vs. MH, respectively) indicating our results are not affected by the use of the averaged brain for our reconstructions.

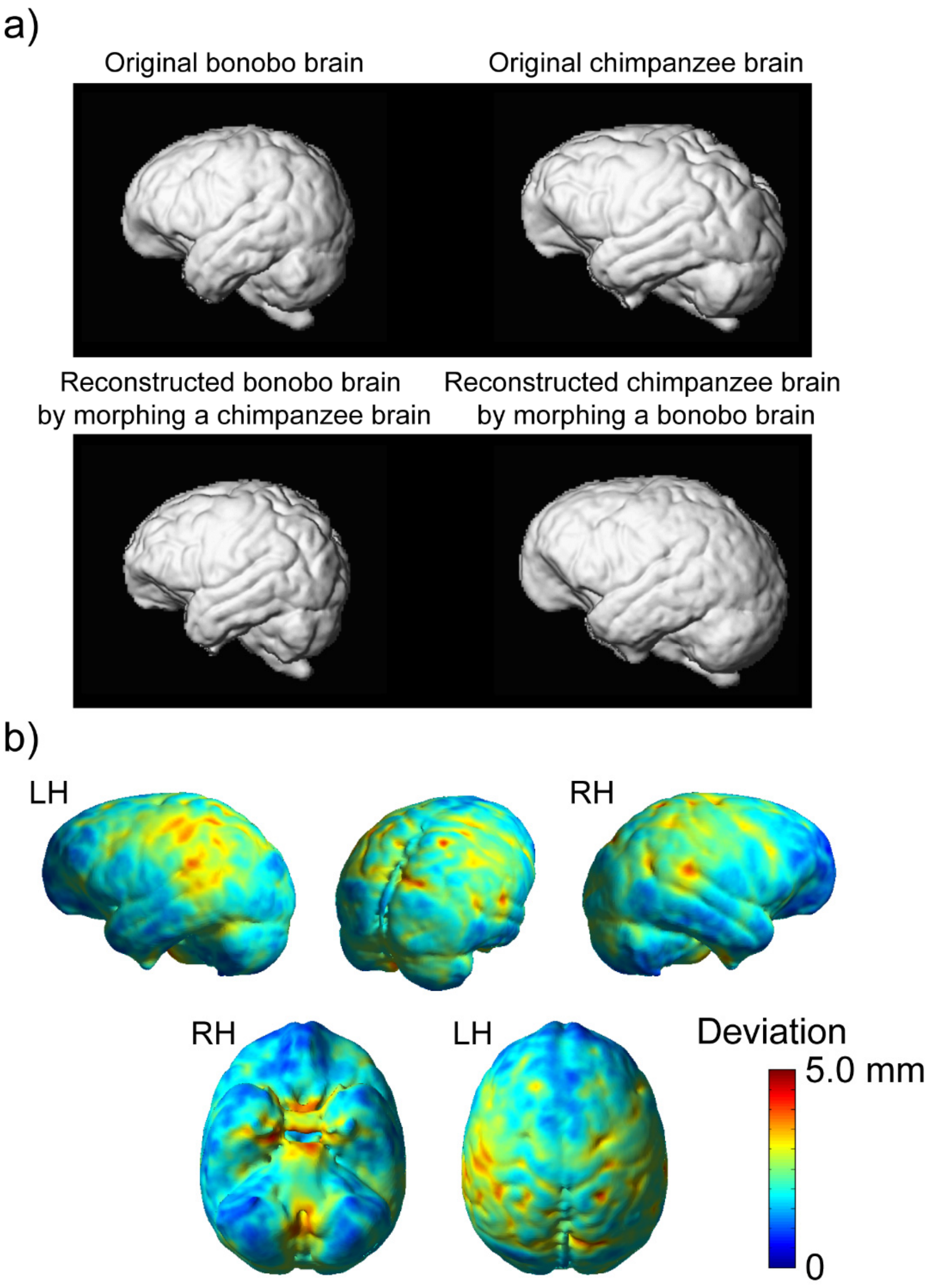

Extended Data Figure 5 | Evaluation of the bonobo (chimpanzee) brain reconstructed by deforming the chimpanzee (bonobo) brain. a, Reconstructed brains compared with the originals (the average brain transformed back to the specific individual using a DARTEL algorithm). **b**, Evaluation of accuracy. The colour map indicates the mean deviation from the true value. The colour map is overlaid on the average brain surface across 8 chimpanzees and 3 bonobos.

Extended Data Table 1 | Correspondence between the AAL atlas and the parcellated brain regions

Region	Subregion	Region name of AAL atlas					Abbrevation
Frontal	Superior / Middle regions	Frontal_Sup	Frontal_Mid	Supp_Motor_Area			Fr SM
	Inferior regions	Frontal_Inf_Oper	Frontal_Inf_Tri				Fr I
	Orbitofrontal regions	Frontal_Sup_Orb	Frontal_Mid_Orb	Frontal_Inf_Orb	Frontal_Med_Orb	Rectus	Fr O
Sensory-motor		Precentral	Postcentral	Paracentralobule			Sm
Parietal	Superior / Inferior regions	Parietal_Sup	Parietal_Inf	Precuneus			Pa SI
	Temporo-parietal junction	SupraMarginal	Angular				Pa TP
Temporal	Superior / Middle regions	Temporal_Sup	Temporal_Mid				Te SM
	Inferior / Medial regions	Temporal_Inf	ParaHippocampal	Temporal_Pole_Sup	Temporal_Pole_Mid		Te I
Occipital	Superior / Middle regions	Calcarine	Cuneus	Occipital_Sup	Occipital_Mid		Oc SM
	Inferior regions	Occipital_Inf	Lingual				Oc I
Cerebellum	Anterior parts	Cerebelum_3	Cerebelum_4_5				Ce A
	Posterior parts	Cerebelum_Crus1	Cerebelum_Crus2	Cerebelum_6	Cerebelum_7b		Ce P
		Cerebelum_8	Cerebelum_9	Cerebelum_10			
	Vermis	Vermis_1_2	Vermis_3	Vermis_4_5	Vermis_6		Ce V
		Vermis_7	Vermis_8	Vermis_9	Vermis_10		

Extended Data Table 2 | Mean accuracy of the estimated volume in each parcellated brain region

Region	Subregion	LH	RH
Frontal	Superior / Middle regions	88.2 ± 2.4	88.6 ± 2.6
	Inferior regions	88.3 ± 2.4	89.9 ± 2.3
	Orbitofrontal regions	92.7 ± 1.2	93.0 ± 1.2
Sensory-motor		83.6 ± 2.8	84.3 ± 2.7
Parietal	Superior / Inferior regions	90.1 ± 1.9	90.1 ± 1.9
	Temporo-parietal junction	92.4 ± 1.7	92.3 ± 1.5
Temporal	Superior / Middle regions	92.6 ± 1.3	91.7 ± 1.5
	Inferior / Medial regions	93.9 ± 1.3	94.1 ± 1.1
Occipital	Superior / Middle regions	92.0 ± 1.4	92.0 ± 1.5
	Inferior regions	94.7 ± 1.3	94.0 ± 1.3
Cerebellum	Anterior parts	94.3 ± 2.0	96.2 ± 1.5
	Posterior parts	95.9 ± 1.1	95.6 ± 1.1
	Vermis	92.0 ± 2.3	

LH, left hemisphere; RH, right hemisphere. Data are means ± s.d.

Extended Data Table 3 | Statistical test results of differences in the relative volumes of the parcellated brain regions among NT, EH and MH.

Region	Subregion	$F_{2,1190}$	p	t_{1190}		
				NT vs. EH	NT vs. MH	EH vs. MH
Frontal	Superior / Middle regions	1.015	0.363			
	Inferior regions	0.181	0.834			
	Orbitofrontal regions	0.252	0.777			
Sensory-motor		3.646	0.026	0.640	1.407	2.310
Parietal	Superior / Inferior regions	9.313	0.000	0.902	**3.626**	**2.353**
	Temporo-parietal junction	0.870	0.419			
Temporal	Superior / Middle regions	2.932	0.054			
	Inferior / Medial regions	2.437	0.088			
Occipital	Superior / Middle regions	8.149	0.000	0.043	**2.890**	**2.829**
	Inferior regions	5.639	0.004	0.796	1.749	**2.873**
Cerebellum	Anterior parts	0.689	0.502			
	Posterior parts	6.699	0.001	**2.331**	**3.644**	0.354
	Vermis	7.339	0.001	**3.413**	**3.642**	1.176

Bold: $p < 0.05$ corrected for multiple comparisons with Ryan's method.
